# A brief review concerning Chimeric Antigen Receptors T cell therapy

**DOI:** 10.7150/jca.46308

**Published:** 2020-07-11

**Authors:** Ling-Lin Li, Hong-Ling Yuan, Yu-Qiong Yang, Lin Wang, Ren-Chao Zou

**Affiliations:** 1 Department of Hepatobiliary Surgery, The Second Affiliated Hospital of Kunming Medical University, Kunming, 650101, Yunnan, China.; 2 Department of Nephrology, The First Affiliated Hospital of Kunming Medical University, Kunming 650032, Yunnan, China.; 3 Department of Nephrology, The Third People's Hospital of Yunnan Province, Kunming, Yunnan, P.R. China.

**Keywords:** Chimeric antigen receptors, Cancer, Complication, Relapse

## Abstract

The understanding concerning the function of immune system in cancer has achieved considerable advance with time passes by. Manipulating genetically engineered immune cells were investigated as a novel strategy for treating cancer. Chimeric antigen receptors (CARs) are recombinant protein molecules by merging the exquisite targeting the potent cytotoxicity of T cells and specificity of monoclonal antibodies and, which could trigger serial cascades of signal transduction and thereby activate T cells to directly destroy the tumor cells. Manufacturing CAR-modified T lymphocytes were successfully implemented in treating cancer derived from they could specifically retarget tumor-associated antigens, causing effective elimination of tumor cells, which spurred the optimization and development of new CAR-T cell technology. The advancement of synthetic biology methodologies of cell therapy in CAR-T would ultimately provide us with a much safer, reliable and efficient modality to against cancer. This review primarily described the emergence, development and application of cell therapy in CAR-T, then discuss the side effects and the potential factors of tumor reccurrence caused by CAR-T cell therapy, in addition to the corresponding countermeasure concerning complications.

## Introduction

As we all known, the immune system was traditionally divided into two main types consisting of innate and adaptive immunity, which can vigorously identify, guard and defend against foreign invaders 1. Typically, innate immunity was activated with an instant and non-specific way involved in various cells including innate lymphoid cells neutrophils, mast cells, natural killer (NK) cells and macrophages cells 2, which could be promptly triggered within minutes to hours through rapid defense mechanisms following pathogens invasion 3. Conversely, the adaptive immune response slowly emerged depending on the initiation and subsequent outcome of innate immune response at several days after undergoing damage. Numerous cells participated in the process of regulating immune response. Dendritic cells (DCs) and macrophages could, as antigen-presenting cells (APCs), secrete cytokines to stimulate immune cells and present T cells with antigens forming major histocompatibility complex (MHC) molecules 4. Moreover, T cells were swiftly activated so that they can modulate many aspects of the immune response. Activated T cells would acquire memory after proliferating and, and thereafter causing migration of effector T cells to the initial antigens binding site 5. Research shows that T cells are critical against genesis and tumor development derived from their unparalleled ability comprising responding to antigens and then formulating memory, allowing a rapid robust response upon a similar antigen invasion in future 6. Accumulated evidences strongly indicated that the crosstalk between T cells and antigen-presenting cells and could effectively protect against the invasion and destroy of malignant cells and foreign pathogens 7. Generally, T cells could rapidly recognize the tumor-associated antigens then kill tumor cells, and long-term protect against tumor recurrence 8. Currently, cancer immunotherapy has drawn widespread attention in recent times, with treatment mainly destroyed the tumor and prevented its recurrence by activating the adaptive immune system of cancer patients. With the relentless march of science and technological progress, cancer immunotherapy had achieved a major breakthrough owing to the use of monoclonal antibodies and development of adoptive T cell therapies 9, 10. Therein, Chimeric antigen receptors (CARs) are synthetic receptors rooted in reprograming T cells by embedding a single chain variable fragment (scFv) ectodomain and an endodomain consisting of CD3 TCR signal in addition to the costimulatory domain 11, which could specifically target antigens and kill tumor cells 12. To date, the CAR-T cell therapies had been applied for treating certain tumors like acute myeloid leukemia 13, gastric cancer 14, and so on. And CAR-T cell therapy had achieved tremendous success in eradicating hematologic malignancies. Here, we reviewed the great potential and bright prospects, and also noted the current issues induced by employing CAR-T cells therapy in tumors treatment.

## The emergence and development of CAR-T cell therapy

In order to protect the organism and against pathogens invasion, the immune system evolve gradually to distinguish self from non-self. To eliminate cancerous cells, immune system would patrol and spot these dangerous cells via certain signals in the cell surface. However, cancer cells are stemmed from the normal cell gene mutations, and they have evolved the unusual ability including immune evasion and suppression. Therefore, incurable cancer has become one of the most massive challenges to all human societies in this world. The emergence of genetically modified T cells initiated a new epoch for cancer therapy. CAR-T cell could effectively recognize, target and destroy cancer cells through a MHC-independent mechanism. The early engineered T cell could recognize intracellular and extracellular antigen in perspective that MHC depend on cloned T cell receptors (TCR) expression. Whereas, many tumors downregulated the expression of MHC to hide from the TCR engineered T cell. Consequently, chimeric antigen receptors (CAR) were employed to strengthen T cell specificity by combining B cell receptor and T cell receptor domains. At present, CAR-T cell therapy has yielded stunning outcomes in patients with hematologic malignancies. For instance, CD19 is a marker that is highly expressed in B cells and B cell-derived malignancies cell surface 15. CD19-directed CAR-T cells (CART19) therapy had contributed to durable and complete remissions in patients with relapsed and refractory B cell malignancies 16, 17. Nevertheless, CAR-T cell therapy is different from inanimate platforms (antibodies or small molecules) on account of cells capability to sense intelligently and the behaviors of response. Many studies have been conducted regarding how to manufacture, manipulate and control these cellular devices. In future, the advancement of synthetic biology strategies of CAR-T cell therapy would ultimately provide us with a much safer, reliable and efficient modality to against cancer.

## Concise overview concerning CAR-T cell therapy

**Figure 1:**
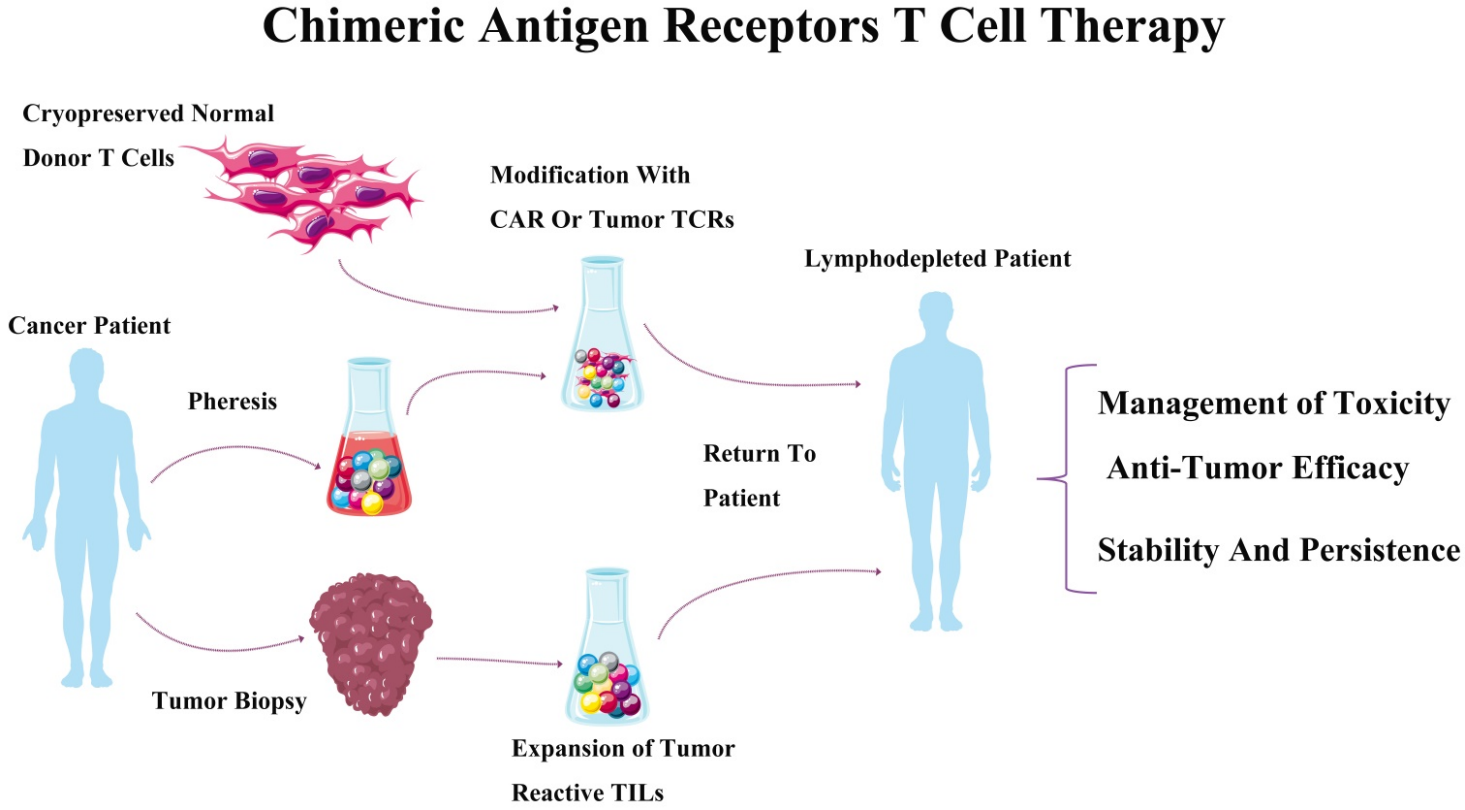
** Chimeric antigen receptors T cell therapy.** The process of chimeric antigen receptors T cell therapy, which mainly includes tumor biopsy, pheresis and expiation, then modification with CAR or tumor TCRs was transfused to tumor patient.

The CAR-T receptor are synthetic transmembrane receptors 18, which are made up of a single chain extracellular antibody fragment (scFv), and intracellular signaling domains from the T Cell Receptor (TCR) and costimulatory molecules 19. CAR-modified lymphocytes have spurred the rapid development for the therapeutically areas of oncology by directing against a broad range of target antigens. CARs could, as recombinant protein molecules, regulate cell activation and redirect cytotoxic lymphocytes toward target antigens including malignant and other target cells 11. The antigen-recognition domain of CARs mainly depends on the sequences of monoclonal antibodies (mAbs) to interact with tumor epitopes in an MHC-unrestricted mode. Briefly, CAR-T cell therapy was described as following: a CAR-encoding DNA cassette was constructed and delivered into primary T cells obtained from a patient. Reprogrammed CAR-T cells were abundantly increased in *ex vivo* environments, and then re-infused into the patient body. Antigen-recognition domain of CAR would activate T cells to destruct the tumor cells when encountered with target tumor cells (**Figure 1**) 20. For now, the high feasibility of CAR-T cell technology implementing in treatment of hematologic malignancies 15, 21 indicated that CAR strategy might be a broadly applicable remedy for cancer 22.

## The essential properties of CAR-T cell therapy

Currently, the genetically engineered CAR-T cell therapy has drawn increasing public attention as a new paradigm of cancer immunotherapy methods. The efficacy, stability and persistence of CAR-T cell *in vivo* were crucial for exerting its anti-tumor activities. These essential properties of CAR-T cell were acquired by using genome editing tools consisting of clustered regulatory interspaced short palindromic repeat, zinc-finger nucleases, and CRISPR-associated protein 9 (CRISPR/Cas9) techniques, and so on 23, 24. These techniques were useful to trace the lineage of CAR-T cell *in vivo* 25, and the functional activity was enhanced and the side effects were managed via the tetracycline regulatory system 26. Meanwhile, the CAR-T cell persistence was enhancing by altering the costimulatory domain 27. Moreover, the anti-tumor efficacy of CAR-T cell was augmented by disrupting programmed cell death protein 1 (PD-1) 28, and the progeny of CAR-T cell through disruption of TET2 also effectively improved the therapeutic efficacy 29.

## The potential mechanisms of CAR-T cell therapy

To deeply illustrate the intricate relationship between tumor cells and immune system would conduce to accelerate the research and development of anti-tumor consisting of rejuvenation of host immunity, activating the immune cells against the cancer cells, and removing the ageing cells. To date, one of the most difficult problems concerning tumor therapy derived from tumor cells escaped from the surveillance of immune system. Generally, tumor cells avoid from cell mediated immunity by changing surface proteins and intracellular signal molecules 30. Extensive research showed that antibodies based allogeneic stem cell transplantation, immunotherapies, and CAR-T cell therapy effectively suppressed the proliferation and survival of tumor cells 31, 32. Epigenetic mechanisms affect the efficacy of CAR-T cell therapy and induce resistance against tumor cells. In addition, a battery of chromatin modifiers were implicated with maintaining proper chromatin structure spatially and temporally. Chromatin states and DNA modifications integrate signal to suppress/active gene expression by acting as the signaling platform 33, and mediate the gene expression pattern 34. Signal transduction pathways also directly cross talk chromatin to change the epigenetic landscape, and the covalent modifications improve the therapeutic outcomes of CAR-T cell. Epigenetic agent administration prevents immune escape of tumor cells, and enhances the anti-tumor activity of CAR-T cell involving in posttranslational regulation 35. Therefore, epigenetic mechanism comprising of chromatin/DNA methylation states and non-coding RNA was further investigated to demonstrate the correlation between tumor prognosis and CAR-T cell therapy.

## Combination of CAR-T cell therapy with other therapeutics

Tumor formation is a gradually evolving process involving in multiple mutations, The CAR-T cell therapy combined with other therapeutics may achieve specific toxicity to effectively inhibit the tumor progression and kill tumor cells 36. Solid tumors are compact and inaccessible to infiltration, and appropriate heat treatment may increase the drug's permeability. Hence, CAR-T cell combined with photothermal therapy may improve the permeability of CAR-T cells 37. Meanwhile, the immune-checkpoint inhibitors play an important role in maintaining the antitumor activity through overcoming T-cell dysfunction 38. The checkpoint receptors including PD-1 and CTLA-4 (cytotoxic T-lymphocyte associated protein 4), are expressed by activating T cells to negatively regulate the cytotoxic activities 39. Ipilimumab (monoclonal antibody against CTLA-4) promote antitumor activityinvolving in effector T cell exhaustion in patients with metastatic melanoma 40. The IFNγ increases the expression of the PD-L1 protein on T cells and tumor cells, which promotes the PD-1 receptor of T cells to inhibit the antitumor immunity 41, and the deletion of PD-1 elevated the antitumor efficacy of CAR-T cells 28. The IFNγ secreted by activated T cell increases the immunosuppressive molecule expression (IDO), whereas IDO inhibitors combined with CAR-T cell enhance its cytotoxic effect 42. Therefore, CAR-T cell therapy combined with PD-1/CTLA-4 antibodies blockade may achieve objective response in cancer treatment. Recently, CAR-T cell combined with chemotherapeutic agents such as carboplatin significantly improved anti-tumor effect for treating ovarian cancer 43. Meanwhile, CAR-T cell combined with local subtherapeutic irradiation also enhanced antitumor efficacy 44. These studies suggested that CAR-T cell therapy combined with chemotherapy or radiation may further improve its treatment efficacy and treating tumor.

## CAR-T cell therapy and its applications

### CAR-T cell therapy in acute myeloid leukemia

Acute myeloid leukemia (AML) is described when abnormal myeloid progenitors in peripheral blood and bone marrow undergo clonal expansion, which inhibits the creation of normal cells of blood. The statistics suggested that AML patients had a dismal prognosis, and the cure rate was 5-15% for elderly patients, and the patients' average survival span was 5-10 months 45. Despite improving the understanding about AML, the recurrence rate and mortality still remain high using traditional chemotherapy 46. The conventional strategies may hit a ceiling for treating AML, and the high toxicity and limited success compel people to exploit new therapeutics. CAR-T cell technology is a promising immunological approach by merging the exquisite aiming at the potent cytotoxicity of T cells and specificity of monoclonal antibodies and, and has been successfully implemented to cure ALL 47, 48. The CAR-T cell triggered a serial cascades of signal transduction and thereafter activated T cells to directly destroy the tumor cells once recognizing the target antigens 49. More recently, CAR-T cell therapy was considered as a likely therapy to cure AML owing to its durable and potent anti-tumor activity 50. Notably, the CAR-T cells could manufacture CAR-T cells with long-term memory following their activation at initial stages, which was conduce to the long-term elimination of tumor cells and prevent tumor relapse. Some clinical trials showed that anti-CD19 CAR-T cells had achieved an impressive outcomes and long-term remissions in patients with AML 21, 51. In addition to anti-CD19 CAR-T cells, CD123-directed CAR-T cells and CD44v6-directed CAR-T cells also acquired good therapeutic efficacy in AML patients 52. Collectively, the researches results highlighted that CAR-T cell therapy had extensive application prospect and theory value for AML.

### CAR-T cell therapy in solid structured tumors

Enormous hindrances wait beyond CARs safe targets identification and its functional design for treating solid tumors. The efficient retention of viability, maintenance of function and homing of CAR-T cell within the immunosuppressive tumor microenvironment could be a problem to be solved imperatively 53.

### Pancreatic cancer

Monoclonal antibodies or checkpoint inhibitors or were utilized to cure prostate cancer derived from it could specifically recognize the prostate stem cell antigen (PSCA) and prostate-specific membrane antigen (PSMA). Therefore, PSCA CAR-T cell was produced in treatment of pancreatic cancer due to the PSCA is overexpressed in initial stages of pancreatic cancer. Meanwhile, 5E5 modified CAR-T-cells administration reduced the growth of tumor and increased the survival rate in mice with pancreatic cancer. Collectively, CAR-T cell therapy had made gratifying achievements in pancreatic cancer.

### Hepatocellular carcinoma

**Figure 2:**
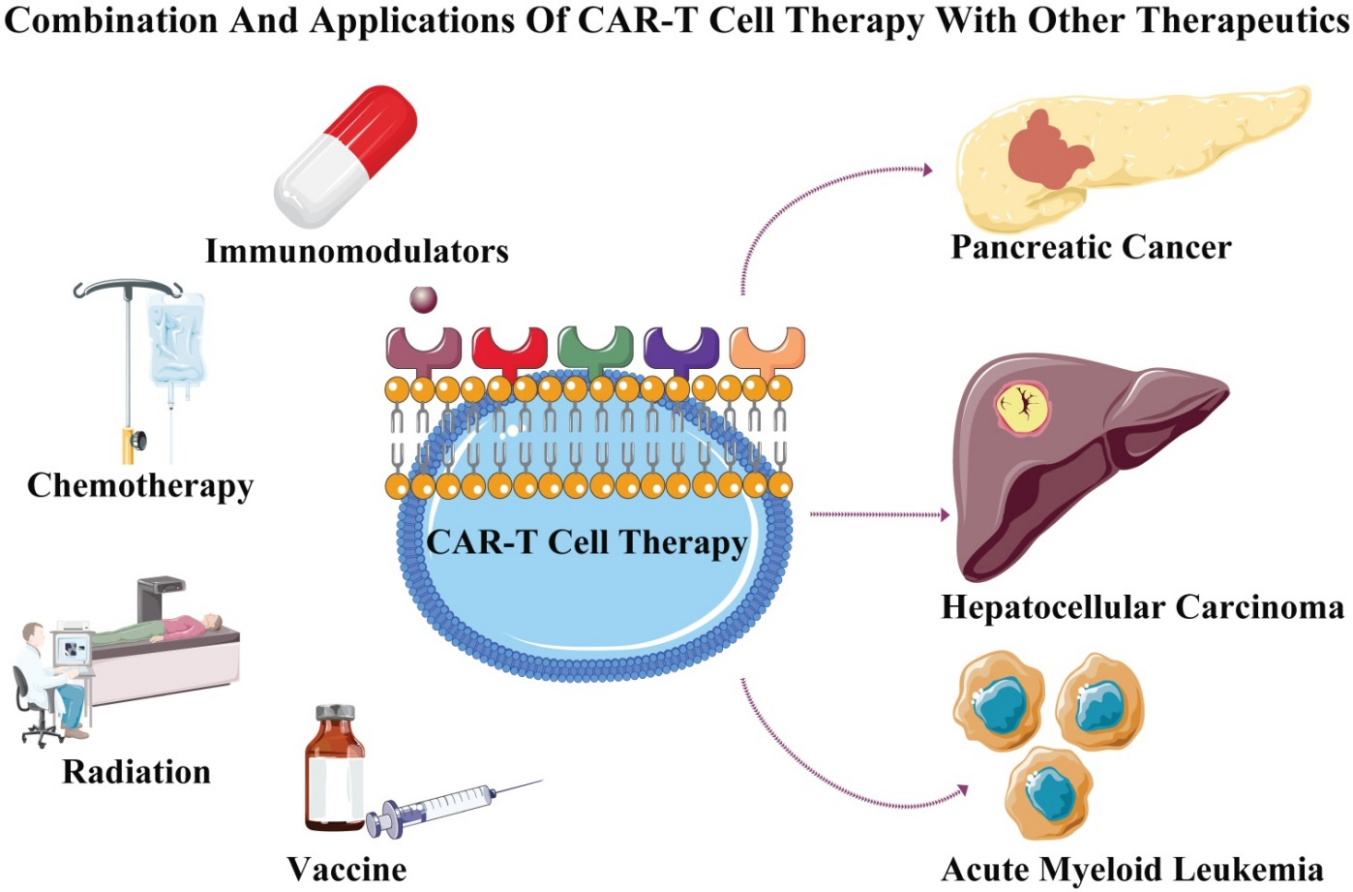
** Combination and applications of CAR-T cell therapy with other therapeutics.** CAR-T cell therapy when separately combined with immunomodulators, chemotherapy, radiation and vaccine to treat tumors consisting of acute myeloid leukemia, pancreatic cancer and hepatocellular carcinoma.

Hepatocellular carcinoma (HCC) is among the most serious malignancies threatening human health, besides being the third leading cause of cancer deaths worldwide 54. Hepatitis B/C (HCV/HBV) viruses are pivotal predisposing factors contributing for approximately 80% of the HCC events. Despite many attempts, the five-year survival rate remained poor, and the majority succumbed to this disease in patients with HCC 55. According to some studies, infiltrating into the tumor site of T and NK cells was closely related to the high survival rate in HCC patients 56. Whereas, immunosuppressive tumor microenvironments including regulatory T cells 57, cancer-associated fibroblasts 58, and tumor associated macrophages 59, have come together to gradually suppress and undermine immune function. Previous researches demonstrated that CAR-T cell composed of CD8α, intracellular signaling domains, 4-1BB, and CD3ζ, the anti-GPC3 scFv, hinge and CD28 transmembrane could specifically lyse HCC cell lines, and significantly suppressing growth in immunodeficient mice, of orthotopic xenografts 60. Furthermore, second-generation CARs (including CD28 costimulation) could specifically target L and HBV S antigens, and thereby enhance the T cells functions to eradicate human hepatoma cells infected with HBV. However, the remedial effect of CAR-T cell was unsatisfactory in HCC, and the inferior efficacy may be due to poor trafficking, hostile tumor microenvironment, penetration of therapeutic cells and target antigen heterogeneity 61. More efforts and attempts would be paid to further investigate the potential strategy for treating HCC by using engineered CAR-T cell (**Figure 2**).

## The side effects caused by CAR-T cell therapy

CARs could, redirect antigen specificity as artificial receptors, and enhance T cell function to target an array of tumor cell surface antigens by means of the costimulatory component 62. CAR-T cell therapy is a novel strategy for treating cancer. Additionally, previous researches indicated that CAR-T cell therapy hold tremendous insights to cancer therapy 63. CAR-T cells infusion would result in cytokine release syndrome (CRS) as a side effect beside off-tumor/on-target toxicity, and so on. CRS is one of the potentially lethal side effects following CAR-T cell therapy. Proliferation and activation of CAR-T cell *in vivo* induced a rapid inflammatory systemic response and then caused dramatic increases of inflammatory cytokines 64, which ultimately resulted in high-grade fevers, respiratory insufficiency, hypotension, and neurologic dysfunction 21. Researches documented that IL-6 participated in constructing a classic feedback loop, with hindrances of the mechanism of IL-6 could halt the toxicity induced by CAR-T cell therapy. CAR-modified T cell derived from murine antibodies provided self-limited expression, while administration by using an intermittent dosing schedule to achieve antitumor effects optimally, ultimately gave raise to anaphylaxis associated with IgE antibody response to CAR 65. A suicide construct for CAR-T cells ablation is a safe high throughput strategy to control adverse events consisting of engraftment that are prolonged and attenuating severe toxicities (Such as CRS). Moreover, the underlying mechanism concerning the other side effects containing macrophage activation syndrome, hepatosplenomegaly (HSM), and low fibrinogen still need to be further investigated.

## Cerebral edema induced by CAR-T cell therapy

In addition to CRS, neurotoxicity characterized by varying the ratios of seizures, cognitive dysfunction and focal neurologic deficits is another obvious side effects following CAR-T cell therapy. Among them, fatal cerebral edema is one of the most serious consequences caused by CAR T-cell therapy. Histopathological findings comprising activated microglia, fragmentation of GFAP and perivascular exudates with fibrin deposition indicated that the secondary cerebral edema induced by CAR-T cell therapy may result from the disruption of the blood-brain barrier (BBB), high cytokine levels and astrocyte dysfunction 66, 67. The concurrent disseminated intravascular coagulation following cerebral edema may derive from the downregulation of fibrinogen and elevation of D-dimer levels. Moreover, the increase of endothelial cell activation, capillary leak, and microvascular permeability may contribute to the severe neurotoxicity and BBB dysfunction. The accumulation of BBB endothelial cells adhesion molecules in response to cytokine exposure may implicate in BBB dysfunction and edema 68. The cytokines (such as TNFa, IL-6, IFNc, and IL-1) overexpression, cytokine-mediated endothelial angiopoietin 1/2 (ANG1/2) signaling, activation and increased BBB permeability and aberrant are crucial in the process of cerebral edema formation 66. Consequently, to further elucidate the underlying mechanism of cerebral edema induced by CAR-T cell therapy is conduced to effectively eliminate tumor cells and minimize the side effects.

## Tumor relapse succeeding CAR-T cell therapy

Current perspective argued that tumor relapse may be result from that CAR-T cell could not recognize antigen-negative cancer cells. Multiple mechanisms participated in the antigen escape-caused relapse. Antigen loss and deleterious mutations in the tumor cells may involve in this process of tumor escape 69. It is feasible by targeting antigens associated to different tumors to elevate the efficiency of CAR-T cell therapy. CARs were redesigned by incorporating costimulatory domains joined to OX-40, CD3ζ, CD137 and CD28, which could effectively enhance cytokine proliferation and production of CAR-T cells, and thereby mediate xenograft models showing tumor regression 70, 71. Thus promoting survival of CAR-T cells and their augmenting potency by co-expressing additional cytokines, ligands and costimulatory receptors were a valid approach to suppress tumor growth, invasion and recidivism 72. Moreover, the immunosuppressive microenvironments are important in the tumor relapse. Inhibitory receptors/pathways contributing to the dysfunction and exhaustion of CAR-T cell may be also one of immune escape mechanisms. Gene-editing technology could increase the efficacy of T-cell therapy by permanently disrupting the negative signaling pathways 73. To solve the relapse cause of antigen escape, CAR-T cell was delicately designed to be triggered by multiple antigens synchronously. For example, a PD-1/TIM-3 receptor segment was incorporated into the CAR-T cell to induce PD-L1/TIM-3 expression within the tumor microenvironment, and thereby augment the granzyme expression, proliferation and cytokine secretion of CAR-T cell 74. Besides, “TRUCK cells” could shape the tumor environment to boost the CAR-T cell therapy efficacy, thereby averting residual cancer cells causes of tumor relapse. More specifically, “TRUCK cells”, as a novel method, could induce IL-12 release, augment T-cell activation, attract and activate cells of innate immunity to target tumor and then eliminate cancer cells 75 (**Table 1**).

## The corresponding countermeasure concerning complications

Currently, gene-editing technology was employed to further optimize the therapy algorithms for CRS. Gene silencing was performed to effectively disturb the expression of CRS-related cytokines in T cells before undergoing transduction. Furthermore, T cell was designed to express a corresponding single chain variable fragment (ScFv), which could specifically block receptor-mediated signal transduction pathway to actively avoid CRS. Moreover, suicide genes were genetically encoded to selectively ablate the adoptively transferred cells, which could effectively prevent collateral damage to contiguous cells and tissues 76. These suicide genes comprising a drug-binding domain cloned into human Caspase9 frame may be help to abrogate the on-target and off-tumor toxicities of CAR-T cell 77. With the rapid development of science and technology, new and greater success will be achieved in overcoming the related complications induced by CAR-T cell therapy.

## Conclusion and Future Perspectives

In the past couple of decades, the CAR-T cell therapy has made great achievements, and entered a new and accelerated phase based on the advances in synthetic biology and genetic engineering. Nevertheless, there are lots of problems need to be solved urgently including security, side effects, and so on. The most concerning issue is that there is no convincing evidence to absolutely confirm the security of CAR-T cells. Admirably, some researchers suggested that CAR-T cell therapy had achieved encouraging clinical outcomes in a subset of patients. Moreover, side effects induced by CAR-T cell therapy deserve further attention. Therefore, to further illuminate the underlying mechanism and optimize design scheme of CAR-T cell would extremely minimize bothersome side effects following CAR-T cell therapy. This review provides useful contents regarding the essential information, application areas, tumor relapse, and side effects of CAR-T cell therapy. In conclusion, the CAR-engineered T cells represent a valuable and attractive therapeutic strategy for cancer immunotherapy.

## Figures and Tables

**Table 1:** The side effects caused by CAR-T cell therapy

Side Effect	Pathogenesis	Symptom	Reference
**Cytokine Release Syndrome**	Dramatic increases of inflammatory cytokines	High-grade fevers, Respiratory insufficiency, Hypotension, Neurologic dysfunction	62-65
**Cerebral Edema**	Blood-brain barrier, High cytokine levels, Astrocyte dysfunction	Seizures, Cognitive dysfunction, Focal neurologic deficits	66-68
**Tumor Relapse**	Antigen loss, Deleterious mutations, Dysfunction and exhaustion of CAR-T cell	Tumor growth, invasion and recidivism	69-75
